# Choroidal Changes in Eyes With Polypoidal Choroidal Vasculopathy After Anti-VEGF Therapy Imaged With Swept-Source OCT Angiography

**DOI:** 10.1167/iovs.62.15.5

**Published:** 2021-12-03

**Authors:** Mengxi Shen, Hao Zhou, Kiyoung Kim, Qiyu Bo, Jie Lu, Rita Laiginhas, Xiaoshuang Jiang, Quan Yan, Prashanth Iyer, Omer Trivizki, Yingying Shi, Luis de Sisternes, Mary K. Durbin, William Feuer, Giovanni Gregori, Ruikang K. Wang, Xiaodong Sun, Fenghua Wang, Seung-Young Yu, Philip J. Rosenfeld

**Affiliations:** 1 Department of Ophthalmology, Bascom Palmer Eye Institute, University of Miami Miller School of Medicine, Miami, Florida, United States; 2 Department of Bioengineering, University of Washington, Seattle, Washington, United States; 3 Department of Ophthalmology, Kyung Hee University Medical Center, Kyung Hee University, Seoul, South Korea; 4 Department of Ophthalmology, Shanghai General Hospital, Shanghai Jiao Tong University School of Medicine, Shanghai, China; 5 Department of Ophthalmology, West China Hospital, Sichuan University, Chengdu, China; 6 Research and Development, Carl Zeiss Meditec, Inc., Dublin, California, United States

**Keywords:** polypoidal choroidal vasculopathy, choroid, OCT angiography, anti-VEGF

## Abstract

**Purpose:**

Swept-source optical coherence tomography angiography was used to investigate choroidal changes and their association with pigment epithelial detachments (PEDs) in eyes with polypoidal choroidal vasculopathy (PCV) after treatment with vascular endothelial growth factor (VEGF) inhibitors.

**Methods:**

Patients with treatment-naïve PCV were included and underwent anti-VEGF therapy. Mean choroidal thickness (MCT), choroidal vascularity index (CVI), and PED volume measurements were obtained before and after treatment.

**Results:**

Thirty-four treatment-naïve PCV eyes from 33 patients were included. The PED volume decreased after treatment (*P* < 0.05). The MCT decreased from 223.0 ± 79.6 µm at baseline to 210.9 ± 76.2 µm after treatment (*P* < 0.001). The CVI at baseline was 0.599 ± 0.024, and the CVI after treatment was 0.602 ± 0.023 (*P* = 0.16). There was a correlation between the decreased PED volumes and the decreased MCT measurements (*r* = 0.47; *P* = 0.006). Also, there was a correlation between the decreased PED volumes and the increased CVI measurements (*r* = −0.63; *P* < 0.001).

**Conclusions:**

In treatment-naïve eyes with PCV, the decreases in PED volumes were correlated with the decrease in MCT and the increase in CVI measurements. We propose that, at baseline, the PCV lesions serve as high-volume arteriovenous shunts between choroidal arterial and venous circulation, causing transudation into the choroidal stroma. We propose that, after treatment, the blood flow through the vascular shunt is reduced, the excess stromal transudation is resorbed, and the exudation from the neovascular lesion is reduced, resulting in thinning of the choroid, resolution of the PEDs, and an increase in the CVI due to the resorption of excess choroidal transudation.

Polypoidal choroidal vasculopathy (PCV) is a subtype of neovascular age-related macular degeneration that is commonly seen in Asians.^1^^,^^2^ PCV is characterized by a branching neovascular network with terminal polypoidal lesions and large serosanguineous pigment epithelial detachments (PEDs).^3^^,^^4^ Alterations in choroidal anatomy and hemodynamics are believed to play an important role in the pathogenesis of PCV.^5^ Clinicopathological studies of PCV have revealed degeneration of the retinal pigment epithelium (RPE)–Bruch's membrane (BM)–choriocapillaris complex, with the inner choroid containing largely dilated venules along with the presence of fibrin filling the lumina of the choroidal vessels and the extravascular spaces.^6^^–^^8^

Advances in imaging technology over the past two decades have shed light on the understanding of changes that occur within the choroid in eyes with PCV. Dilated choroidal vessels and choroidal vascular hyperpermeability have been observed with indocyanine green angiography (ICGA).^9^ Focal or diffuse increases in choroidal thickness, along with attenuation of the inner choroid, have also been widely documented through the use of optical coherence tomography (OCT).^10^ More recently, OCT angiography (OCTA) has been useful in characterizing these lesions, and swept-source OCTA (SS-OCTA), compared with spectral-domain OCTA, has improved lesion detection and allowed for better visualization of the polypoidal lesions, the branching neovascular networks, and the choroid.^11^^–^^13^ However, in most of the studies, the structure of the choroid has been investigated by using B-scans passing through the foveal region,^14^ so choroidal measurements have been restricted to the subfoveal region without considering the entirety of the macular region. In addition, these measurements of choroidal parameters in eyes with PCV have been limited by the signal attenuation that occurs when imaging the choroid below large PEDs, which limits accurate measurements of choroidal thickness and vascularity.^15^ For this reason, the longer laser wavelength of SS-OCT compared with SD-OCT should provide better imaging of the choroid, but limitations still exist.

To better understand choroidal features in eyes with PCV, we investigated the choroidal changes that occurred in treatment-naïve eyes with PCV before and after treatment with inhibitors of vascular endothelial growth factor (VEGF) using validated SS-OCT algorithms that we developed to automatically segment the choroid and choroidal vasculature in order to obtain measurements of mean choroidal thickness (MCT) and the choroidal vascularity index (CVI).^16^ We undertook this study of choroidal changes in eyes with PCV after anti-VEGF therapy with the goal of correlating the changes in exudation and PED volumes with changes in the choroid to provide evidence that might support our proposed hemodynamic model of PCV.

## Methods

Patients with treatment-naïve PCV imaged during routine clinical care at Kyung Hee University Medical Center and Shanghai General Hospital from April 2017 to May 2020 were included. This study was performed in accordance with the tenets of the Declaration of Helsinki and the Health Insurance Portability and Accountability Act of 1996, and it was approved by the institutional review boards at both institutions. Informed consent was obtained from all patients.

SS-OCTA imaging (PLEX Elite 9000; Carl Zeiss Meditec, Dublin, CA), using a central wavelength of 1050 nm and a scanning rate of 100,000 A-scans per second, was performed at the baseline and at follow-up visits after anti-VEGF injections. The SS-OCTA images at baseline and after consecutive anti-VEGF injections were selected for analysis. In order to correlate changes in the eye with PCV before and after treatment, the time between the last injection and the date of post-treatment SS-OCTA imaging did not exceed 2 months. All eyes were imaged with the 6 × 6-mm scan pattern. This scan pattern consisted of 500 A-scans per B-scan at 500 B-scan positions (with each B-scan generated from two repetitions), resulting in a uniform spacing of 12 µm between adjacent A-scans in a 6 × 6-mm field of view.

The diagnosis of PCV was based on the first visit when SS-OCTA imaging was acquired by using a slab with segmentation boundaries along the RPE and Bruch's membrane (RPE-fit).^17^ When using the RPE to RPE-fit slab, we reviewed the en face angiographic and structural images, as well as the corresponding B-scans, to detect both the flow and structural profiles consistent with a PCV lesion. Polypoidal lesions were recognized as flow signals underlying a sharp, peaked PED and sub-RPE ring-like lesions on B-scans, described as a round structure seen under the PED with a hyporeflective center and hyperreflective outline.^3^ In addition, polypoidal lesions were identified as having a nodule-like configuration on en face images corresponding to previously observed features using en face ICGA imaging.^18^ Branching neovascular networks, or the type 1 macular neovascularization (MNV) associated with the polyps, were recognized as vascular patterns on en face flow and structural images corresponding to the double-layer sign with a flow signal on the cross-sectional B-scans. Type 2 MNV was defined as a flow signal above RPE on the cross-sectional B-scans and on en face images when the outer slab boundary was at the RPE. At Kyung Hee University Medical Center, the diagnosis of PCV was also confirmed with the use of ICGA imaging. The anti-VEGF treatment was initiated when symptomatic intraretinal fluid (IRF), subretinal fluid (SRF), or serous PED was identified on OCT. Exclusion criteria included low-quality SS-OCTA images with a signal strength of less than 7, motion artifacts, shadowing due to media opacities, massive hemorrhages that obscured detection of the PCV lesion, high myopia (≥6.00 diopters [D]), previous vitrectomy, and the presence of other concomitant retinal diseases. Snellen visual acuity testing was performed at each visit. Snellen visual acuity measurements were converted to approximate Early Treatment Diabetic Retinopathy Study (ETDRS) letter scores for statistical manipulations.^19^

The volumetric measurements of the PEDs were obtained from the 6 × 6-mm scans. PED volumes were obtained using an algorithm that segmented the RPE and BM as previously described and validated based on the volumetric and area assessments of drusen.^20^ When the lesions had been segmented on the instrument and any segmentation errors were edited on the instrument to ensure appropriate boundary segmentation, the processed OCT datasets were exported from the instruments and uploaded to the Advanced Retina Imaging (ARI) Network Hub, a cloud-based processing service for SS-OCTA data provided by the instrument manufacturer (Carl Zeiss Meditec). The PED volume was calculated in the ARI Network Hub using version 10 of the Advanced RPE Analysis algorithm that was previously validated for drusen; this algorithm is also available on the latest software version (2.1) on the instrument. This algorithm used the edited RPE and BM segmentations from the instrument as the two segmentation boundaries to measure the PED area and volume and also to create en face color-coded PED volume maps. Square-root transformations of area measurements and cube-root transformations of volume measurements were performed to eliminate the impact of lesion size on the variability of the standard deviations previously determined from test–retest measurements. The advantages provided by using these transformations have been described previously.^21^

**Figure 1.**
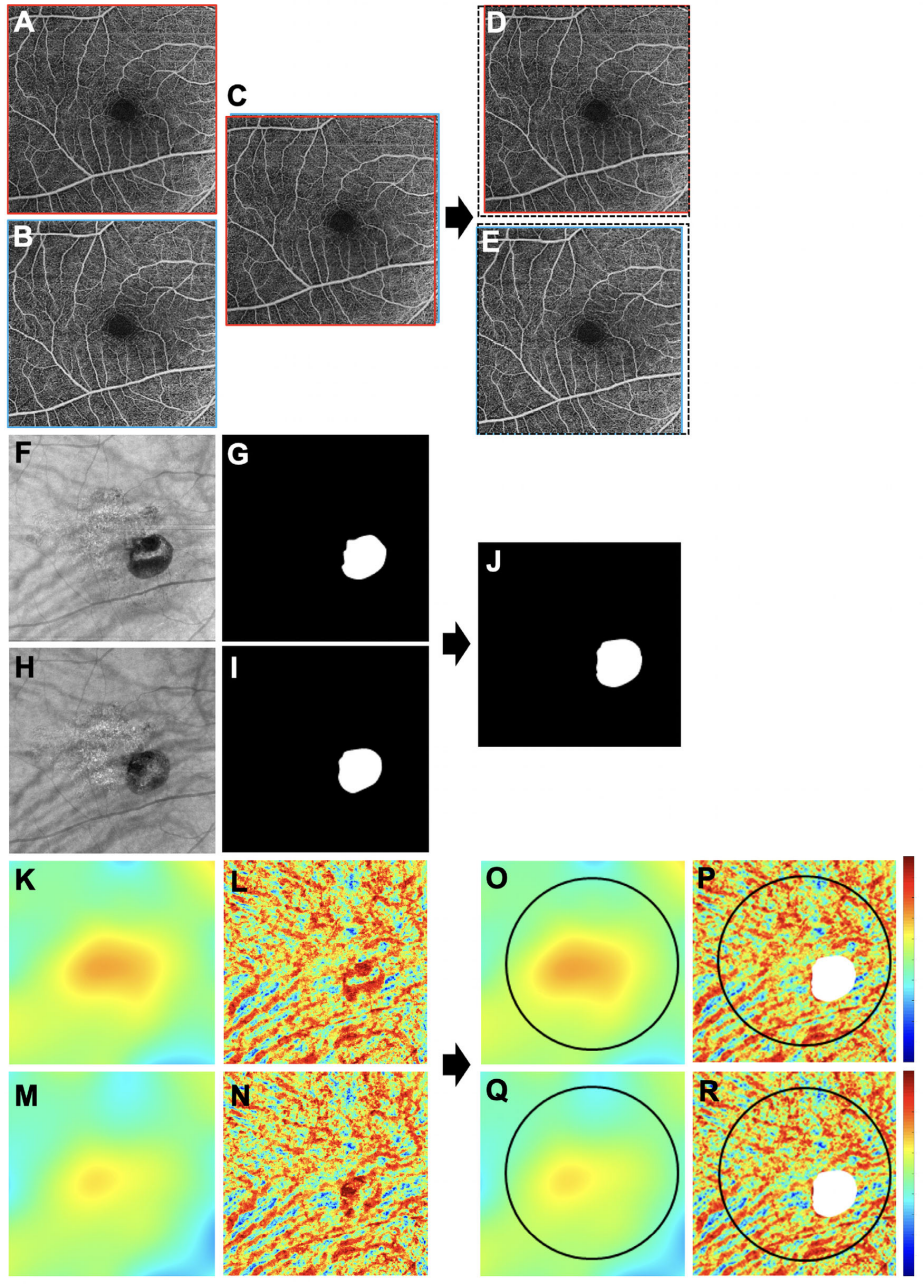
Schematic describing the process for obtaining the measurements of MCT and CVI before and after treatment. (**A**–**E**) Scans were registered using en face retina OCTA images from the two visits. (**A**, **B**) Original 6 × 6-mm en face retina OCTA images at baseline and after treatment. (**C**) En face retina OCTA images from the two visits registered based on the large retinal vessels to make sure the same area was compared before and after treatment. (**D**, **E**) The cropped images after registration at baseline and after treatment; the *black dotted frames* indicate the original 6 × 6-mm field of view. (**F**–**J**) Retinal PED masks were generated automatically using the thresholding strategy described in the Methods section that was used to exclude the areas causing attenuation of the choroidal signal from the PED. (**F**, **H**) OCT en face images using a 200-µm sub-BM slab at baseline and after treatment. Attenuation of the signal in the choroid from the PEDs appears *dark*. (**G**, **I**) *White regions* represent PED masks at baseline and after treatment. (**J**) PED masks from the two visits were combined to result in a final exclusion area so that the same region was compared between visits. (**K**–**R**) Measurements of MCT and CVI. (**K**, **M**) MCT maps at baseline and after treatment. (**L**, **N**) CVI maps at baseline and after treatment. Note the artifacts caused by attenuation of the choroidal signal from the overlying PEDs. (**O**, **Q**) Measurements of MCT within the 5-mm circle centered on the fovea at baseline and after treatment. (**P**, **R**) Measurements of CVI within the 5-mm circle centered on the fovea at baseline and after treatment. The *white area* represents the combined PED mask that was used to exclude the regions from the CVI measurements because choroidal vessels could not be clearly detected due to attenuation of the choroidal signal from the PED. *Color bars*: 0 to 500 µm for MCT; 0 to 1 for CVI.

MCT and CVI measurements were obtained from the structural portion of the 6 × 6-mm SS-OCTA scans using a strategy previously reported by Zhou et al.^16^^,^^22^ The choroidal measurements were performed in regions that included both the polypoidal lesions and branching neovascular networks. Optical attenuation correction was applied to the OCT scans to eliminate the shadowing effect from retinal layers and to enhance the contrast of the choroidal layer, particularly the choroidal/scleral interface. BM and the choroidal/scleral interface were segmented automatically, and color-coded en face choroidal thickness maps were generated based on these segmentations. MCT was calculated as the mean value of the choroidal thickness within a 5-mm-diameter circular region centered on the fovea. Eyes were excluded from the analyses if more than 10% of the lesion was outside the 5-mm circle centered on the fovea. Choroidal vessels were segmented from the entire choroidal slab using Otsu's global thresholding method. En face CVI maps were generated by calculating the choroidal vessel component of each A-scan position, which is the ratio of the number of pixels that belong to a choroidal vessel to the number of pixels that belong to the choroid slab in each A-scan. The CVI was calculated as the ratio of the choroidal vessel volume to the total choroidal volume within the same 5-mm circle centered on the fovea. As shown in Figure 1, to make sure the same area was compared before and after treatment, en face images from the two visits were registered. Due to signal blockage resulting from PEDs on the underlying choroid, the area where choroidal vessels could not be clearly detected were excluded from the CVI measurements. These areas that were excluded were automatically generated from the OCT en face image using a 200-µm sub-BM slab and applying a thresholding limit to represent the region with low signal intensity. The thresholding limit was determined as (mean – 2 × SD) of signal intensities of 10 typical PEDs on en face images using the 200-µm sub-BM slab. The areas identified in the two visits were combined to obtain a final combined area of exclusion in which the same region was compared between visits. Eyes where the combined excluded area within the 5-mm circle was more than 10% of the area of the circle were not included in further analysis.

Statistical analyses were performed using SPSS Statistics 25 (IBM, Armonk, NY, USA). Data were summarized with mean and standard deviation. The statistical significance of differences between means was assessed with the paired two-sample *t*-test. Mann–Whitney tests were used to assess the difference in MCT and CVI between eyes with a type 2 MNV component and those without. Pearson's correlation coefficient was used to evaluate the relationships among visual acuity, PED area, PED volume, MCT, and CVI. *P* < 0.05 was considered statistically significant. The schematic drawing was created with BioRender.com and Procreate.

## Results

A total of 43 eyes from 42 patients with treatment-naïve PCV receiving anti-VEGF therapy who underwent SS-OCTA imaging between April 2017 to May 2020 were reviewed for eligibility in this study. Of these, six eyes were excluded due to motion artifacts, one eye was excluded due to 162-day interval between the last injection and the after-treatment SS-OCTA imaging (>2 months), and 2 eyes were excluded due to more than 10% of the lesion being outside the 5-mm circle centered on the fovea. For CVI measurements, four more eyes were excluded due to signal attenuation that resulted from the PEDs which prevented clear visualization of the choroidal vessels as described above.

A total of 34 treatment-naïve PCV eyes from 33 patients were included in the analysis. Twenty-one out of 33 patients (64%) were men. The mean age of patients was 67.1 ± 7.3 years (range, 55–85). The mean follow-up time was 2.9 ± 1.4 months (range, 0.6–6.7). The mean number of anti-VEGF injections between the two SS-OCTA imaging visits was 2.5 ± 1.0 (range, 1–5). The mean time between the last injection and after-treatment SS-OCTA imaging was 27 ± 14.6 days (range, 6–57). Patients were treated with ranibizumab, aflibercept, and conbercept. In total, 23 injections of ranibizumab were given among seven eyes, 43 injections of aflibercept were given among 18 eyes, and 20 injections of conbercept were given among nine eyes. Although there might be a difference in the choroidal response based on the type of anti-VEGF drug used, we decided to focus on the class of drugs and elected not to divide the patient population into smaller subgroups for the purpose of comparing the drugs. The baseline mean ETDRS letter score was 60.5 ± 17.3, which improved to 69.2 ± 15.2 letters after treatment. There was an improvement in vision of 8.8 ± 8.5 letters (*P* < 0.001) at the after-injection SS-OCT imaging visit. Both the mean PED area and the PED volume measurements decreased after treatment (all *P* < 0.05) (Table 1). The MCT (*n* = 34 eyes) at baseline was 223.0 ± 79.6 µm, and the MCT after treatment was 210.9 ± 76.2 µm; this change before and after anti-VEGF therapy was significant (*P* < 0.001). The CVI (*n* = 30 eyes) at baseline was 0.599 ± 0.024, and the CVI after treatment was 0.602 ± 0.023; this increase in CVI after anti-VEGF therapy was not statistically significant (*P* = 0.16).

**Table 1.** Characteristics of Subjects With PCV at Baseline and After Treatment

	Mean (SD)		
Characteristics	Baseline	After Treatment	*P* ^*^
BCVA (ETDRS letters)	60.5 (17.3)	69.2 (15.2)	<0.001
PED area (mm^2^)	3.24 (2.45)	2.69 (1.86)	0.014
PED area square root (mm)	1.68 (0.66)	1.53 (0.60)	0.006
PED volume (mm^3^)	0.47 (0.65)	0.27 (0.34)	0.029
PED volume cube root (mm)	0.67 (0.28)	0.56 (0.23)	0.001
MCT (µm)	223.0 (79.6)	210.9 (76.2)	<0.001
CVI	0.599 (0.024)	0.602 (0.023)	0.16

BCVA, best-corrected visual acuity.

* Two-sample *t*-test.

**Figure 2.**
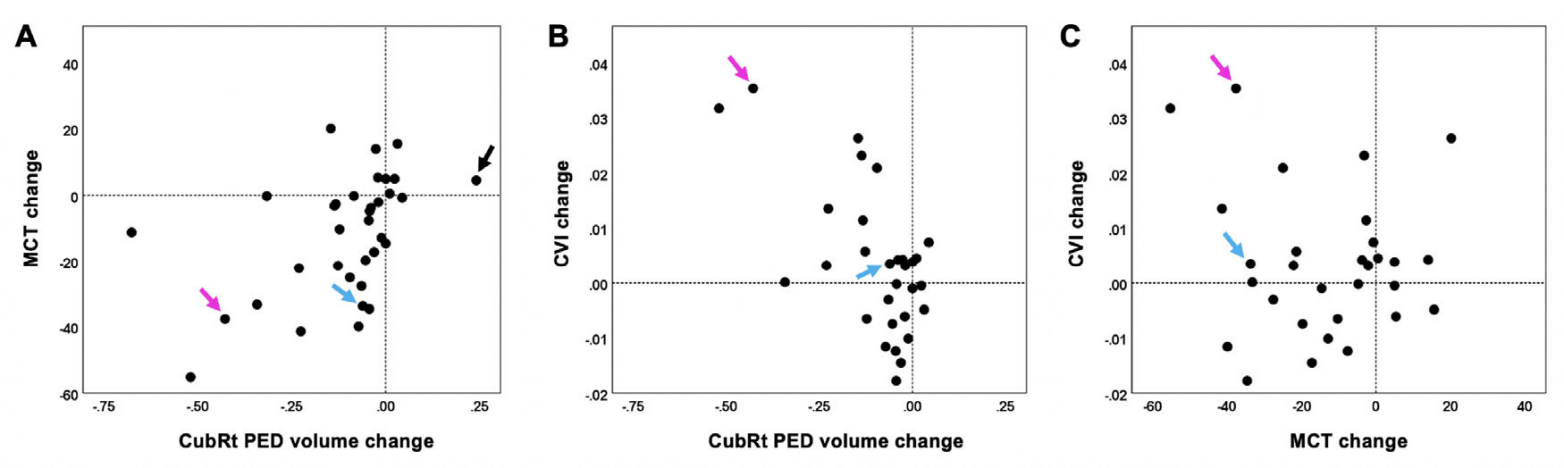
Correlations between changes in retinal PED volume, MCT, and CVI measurements. (**A**) A significant correlation was found between the decrease in PED volumes and the decrease in MCT measurements (*n* = 34; *r* = 0.47; *P* = 0.006). (**B**) A significant correlation was found between the decrease in PED volumes and the increase in CVI measurements (*n* = 30; *r* = −0.63; *P* < 0.001). (**C**) No significant correlation was found between the changes in MCT and CVI measurements (*n* = 30; *r* = −0.11; *P* = 0.56). The *blue arrow* indicates the eye shown in Figures 3 and 4. The *pink arrow* indicates the eye shown in Figures 5 and 6. The *black arrow* indicates an eye with an increase in PED volume shown in Figures 7 and 8. This eye was excluded from CVI measurement.

**Figure 3.**
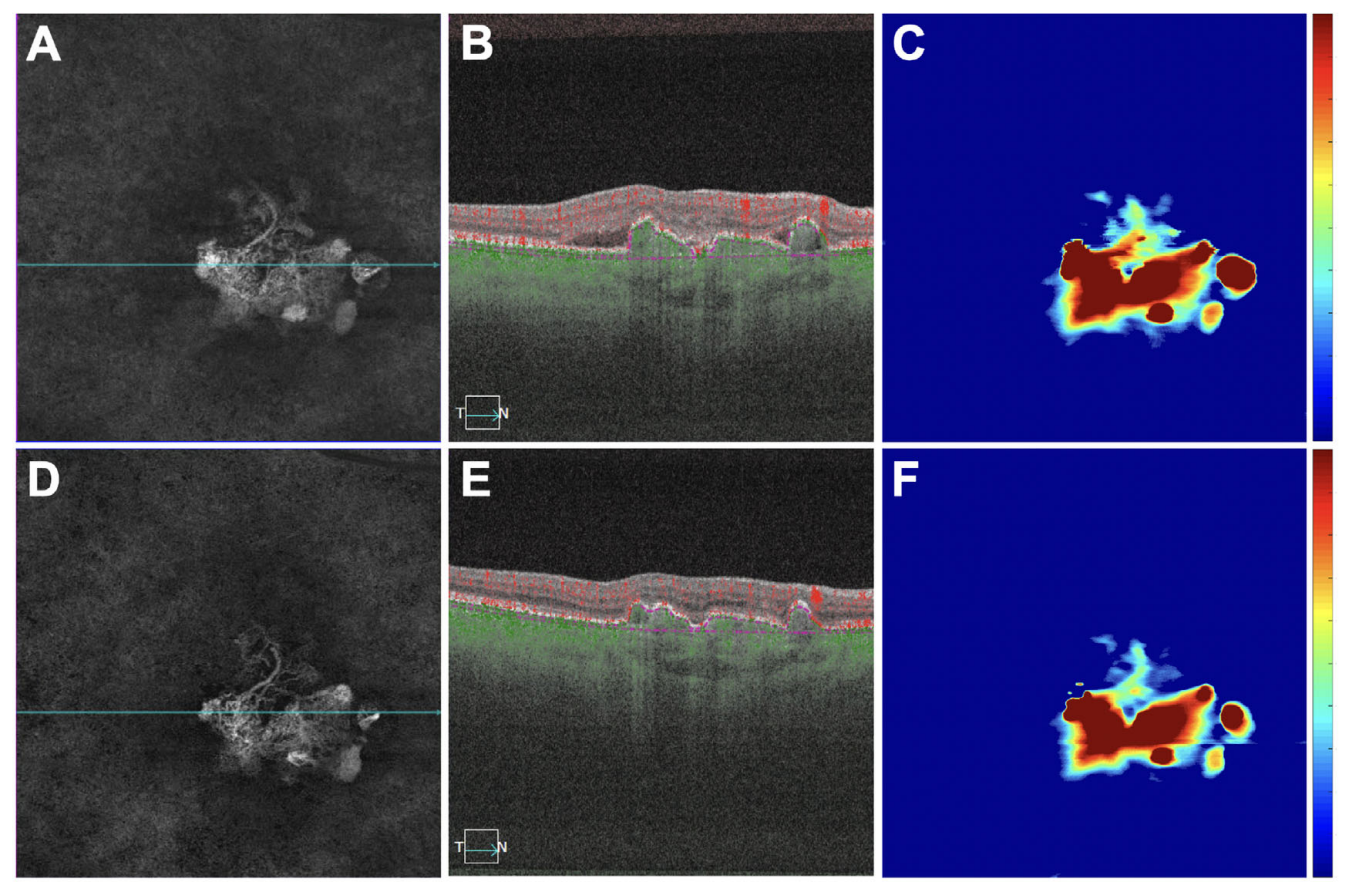
An eye with a decrease in retinal PED volume, a decrease in MCT measurement, and an increase in CVI measurement after anti-VEGF therapy. (**A**–**C**) Images are from the baseline visit before any treatment. (**D**–**F**) Images are 3 months after the baseline visit and after three monthly anti-VEGF injections. (**A**, **D**) En face flow images of the PCV lesion at baseline and after treatment. (**B**, **E**) B-scans with flow corresponding to the *blue lines* in **A** and **D**. (**C**, **F**) PED volume maps at baseline and after treatment. *Color bars:* 0 to 100 µm. PED area = 3.85 mm^2^; PED volume = 0.33 mm^3^ at baseline; PED area = 3.36 mm^2^; PED volume = 0.25 mm^3^ after treatment.

There was a correlation between the decrease in PED volume and the decrease in MCT measurements (*r* = 0.47; *P* = 0.006) (Fig. 2A). There was also a correlation between the decrease in PED volume and the increase in CVI measurements (*r* = −0.63; *P* < 0.001) (Fig. 2B). However, in this study population, there was no overall correlation between MCT and CVI measurements (Fig. 2C). Figures 3^4^,^5^ to 6 show two representative eyes with decreased PED volumes, decreased MCT measurements, and increased CVI measurements after anti-VEGF injections. Although most of the eyes responded well after anti-VEGF therapy, one eye in our current cohort had an enlargement of the PED after three consecutive monthly injections, and the MCT increased, as well (Figs. 7, 8).

**Figure 4.**
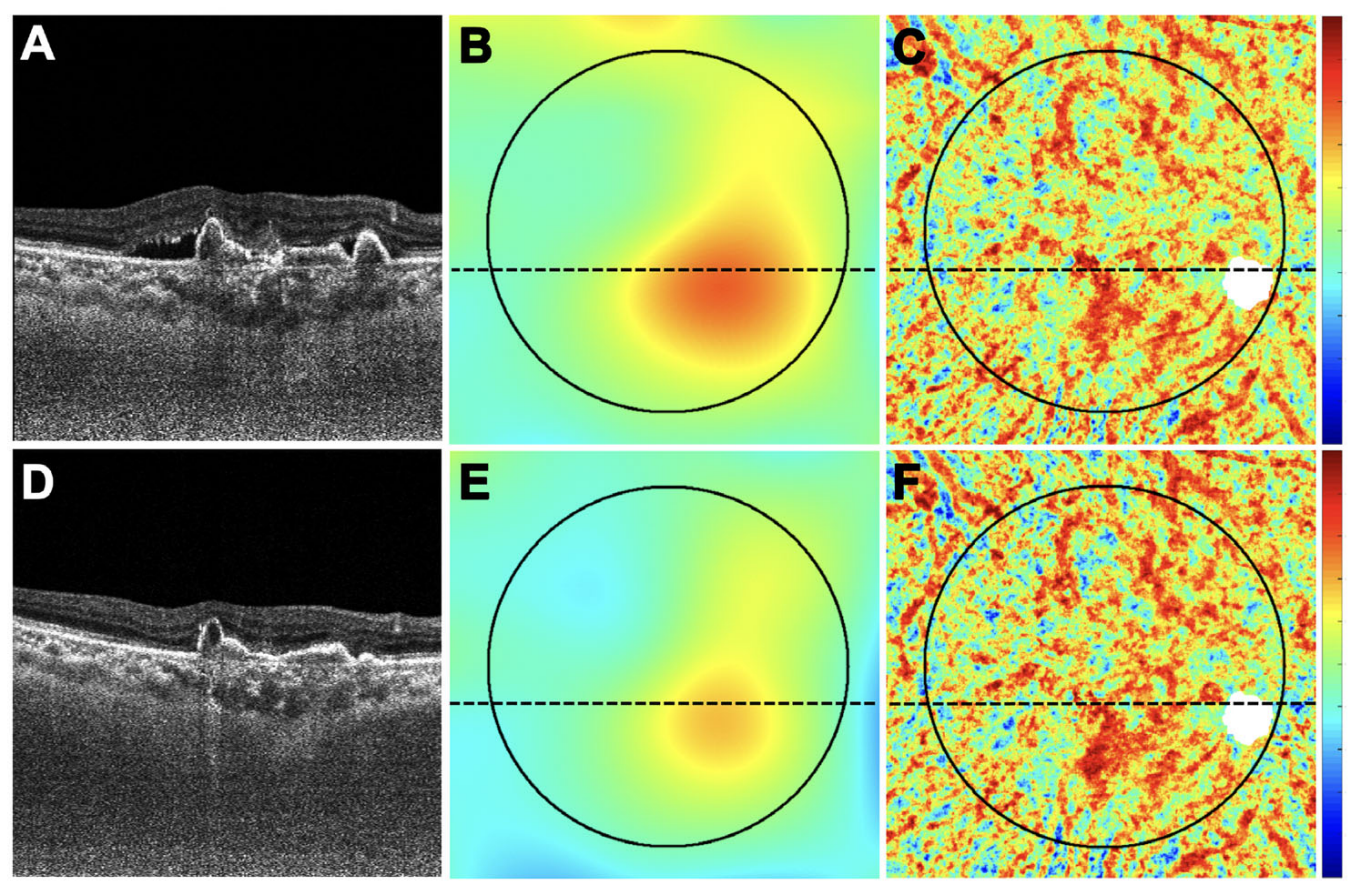
The same eye in Figure 3 with a decrease in retinal PED volume, a decrease in MCT measurement, and an increase in CVI measurement after anti-VEGF therapy. (**A**–**C**) Images are from the baseline visit before any treatment. (**D**–**F**) Images are 3 months after the baseline visit and after three monthly anti-VEGF injections. (**A**, **D**) B-scans after optical attenuation correction, corresponding to the dotted lines in **B**, **C**, **E**, and **F**. (**B**, **E**) MCT maps at baseline and after treatment. The 5-mm circle centered on the fovea is shown that was used to analyze the choroidal measurements from these scans. *Color bar:* 0 to 500 µm. MCT = 278.39 µm at baseline; MCT = 244.73 µm after treatment. (**E**, **J**) CVI maps at baseline and after treatment. *White mask* indicates the area that was excluded from the CVI measurements due to attenuation of the choroidal signal from the PED. *Color bars:* 0 to 1. CVI = 0.629 at baseline; CVI = 0.632 after treatment.

**Figure 5.**
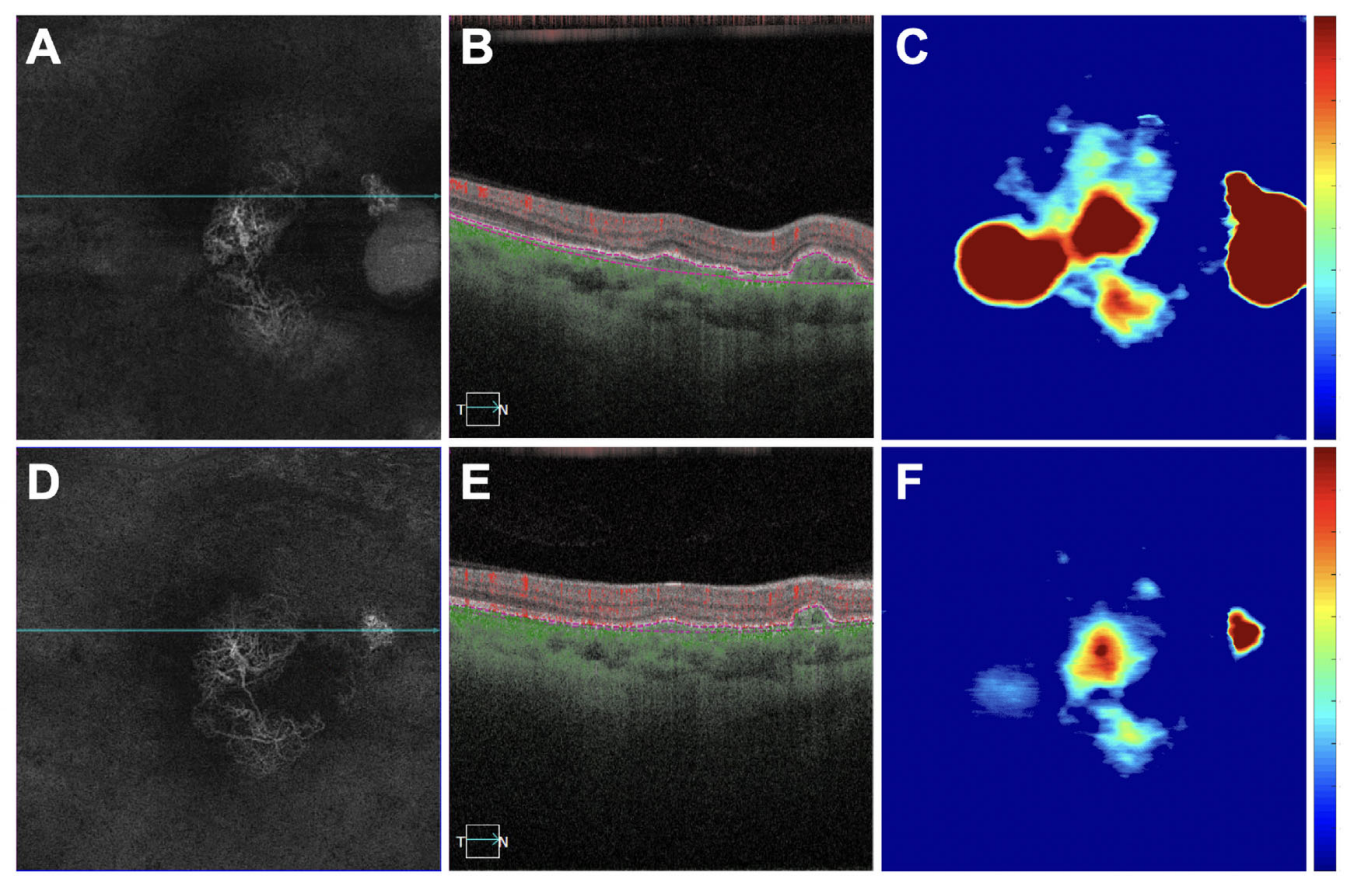
An eye with a decrease in retinal PED volume, a decrease in MCT measurement, and an increase in CVI measurement after anti-VEGF therapy. This is the only eye without any macular fluid at baseline. (**A**–**C**) Images are from the baseline visit before any treatment. (**D**–**F**) Images are 1 month after the baseline visit and after one anti-VEGF injection. (**A**, **D**) En face flow images of the PCV lesion at baseline and after treatment. (**B**, **E**) B-scans with flow corresponding to the *blue lines* in **A** and **D**. (**C**, **F**) PED volume maps at baseline and after treatment. *Color bars:* 0 to 100 µm. PED area = 8.26 mm^2^; PED volume = 0.85 mm^3^ at baseline; PED area = 3.35 mm^2^; PED volume = 0.14 mm^3^ after treatment.

**Figure 6.**
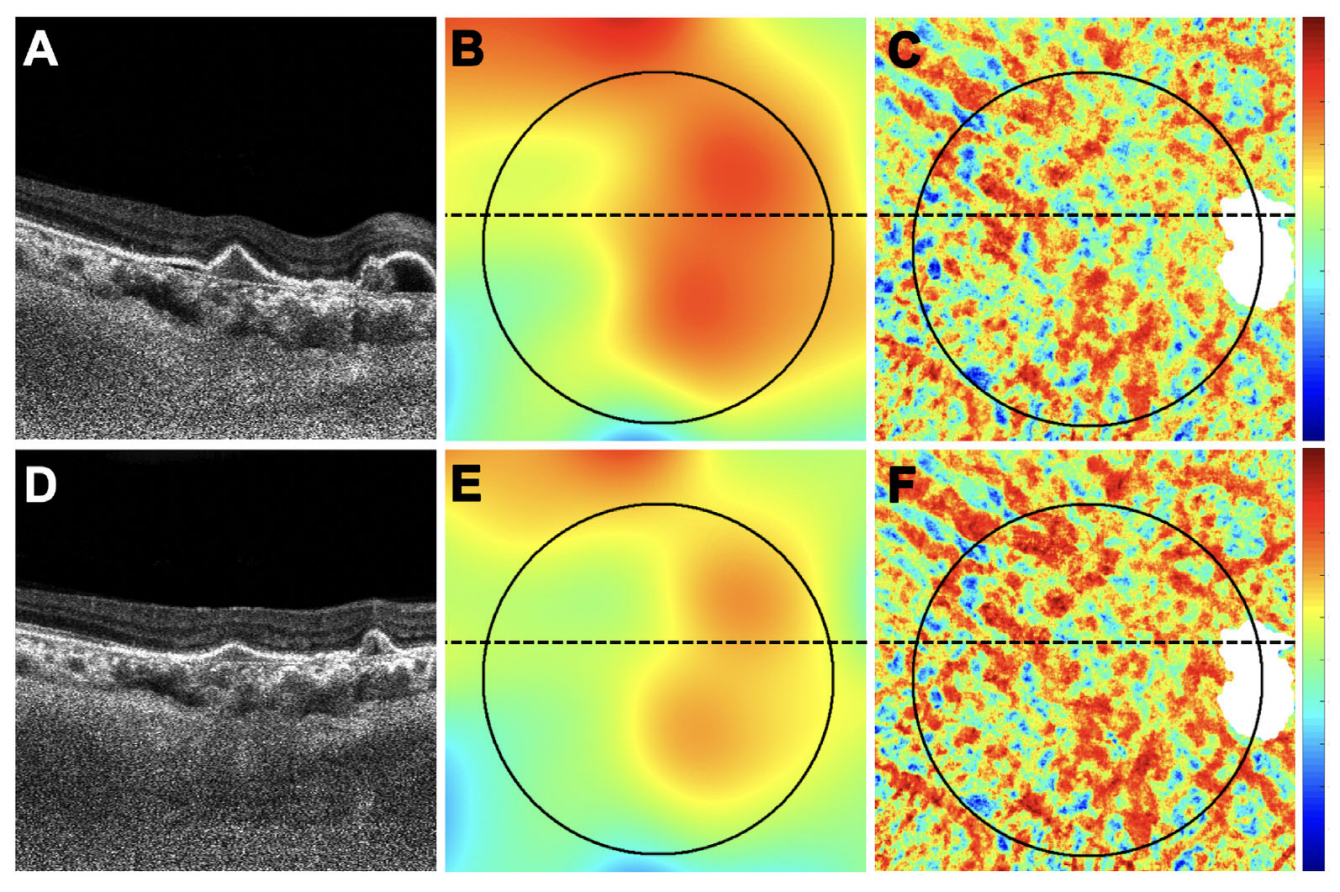
The same eye in Figure 5 with a decrease in retinal PED volume, a decrease in MCT measurement, and an increase in CVI measurement after anti-VEGF therapy. This is the only eye without any macular fluid at baseline. (**A**–**C**) Images are from the baseline visit before any treatment. (**D**–**F**) Images are 1 month after the baseline visit and after one anti-VEGF injection. (**A**, **D**) B-scans after optical attenuation correction, corresponding to the dotted lines in **B**, **C**, **E**, and **F**. (**B**, **E**) MCT maps at baseline and after treatment. The 5-mm circle centered on the fovea is shown that was used to analyze the choroidal measurements from these scans. *Color bar:* 0 to 500 µm. MCT = 330.26 µm at baseline; MCT = 292.68 µm after treatment. (**C**, **F**) CVI maps at baseline and after treatment. *White mask* indicates the area that was excluded from the CVI measurement due to attenuation of the choroidal signal from the PED. *Color bar:* 0 to 1. CVI = 0.61 at baseline; CVI = 0.65 after treatment.

**Figure 7.**
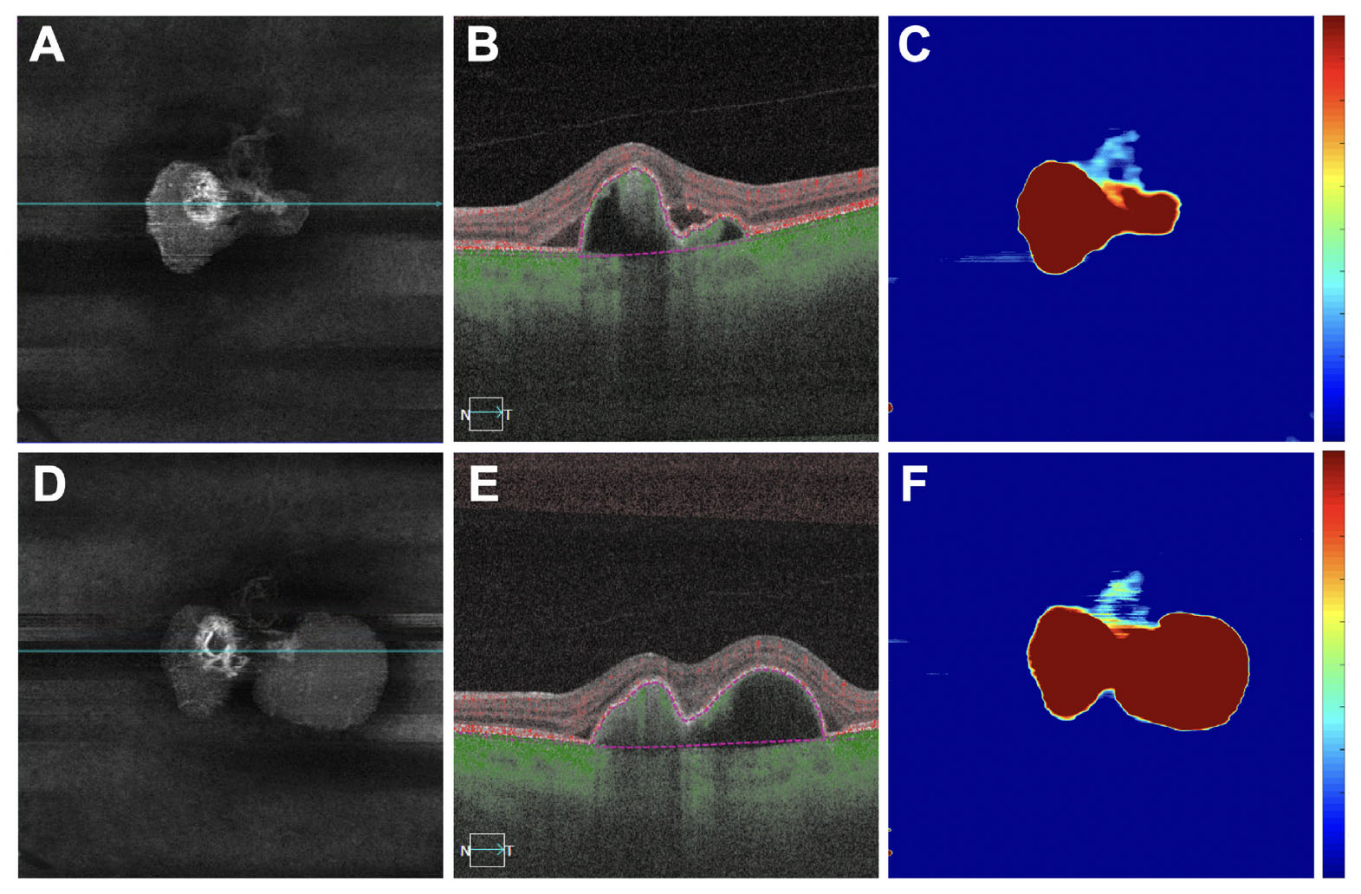
An eye with an increase in retinal PED volume and an increase in MCT measurement after anti-VEGF therapy. (**A**–**C**) Images are from the baseline visit before any treatment. (**D**–**F**) Images are 3 months after the baseline visit and after three monthly anti-VEGF injections. (**A**, **D**) En face flow images of the PCV lesion at baseline and after treatment. (**B**, **E**) B-scans with flow corresponding to the *blue lines* in **A** and **D**. (**C**, **H**) PED volume maps at baseline and after treatment. *Color bars:* 0 to 100 µm. PED area = 2.85 mm^2^; PED volume = 0.70 mm^3^ at baseline; PED area = 4.67 mm^2^; PED volume = 1.44 mm^3^ after treatment.

**Figure 8.**
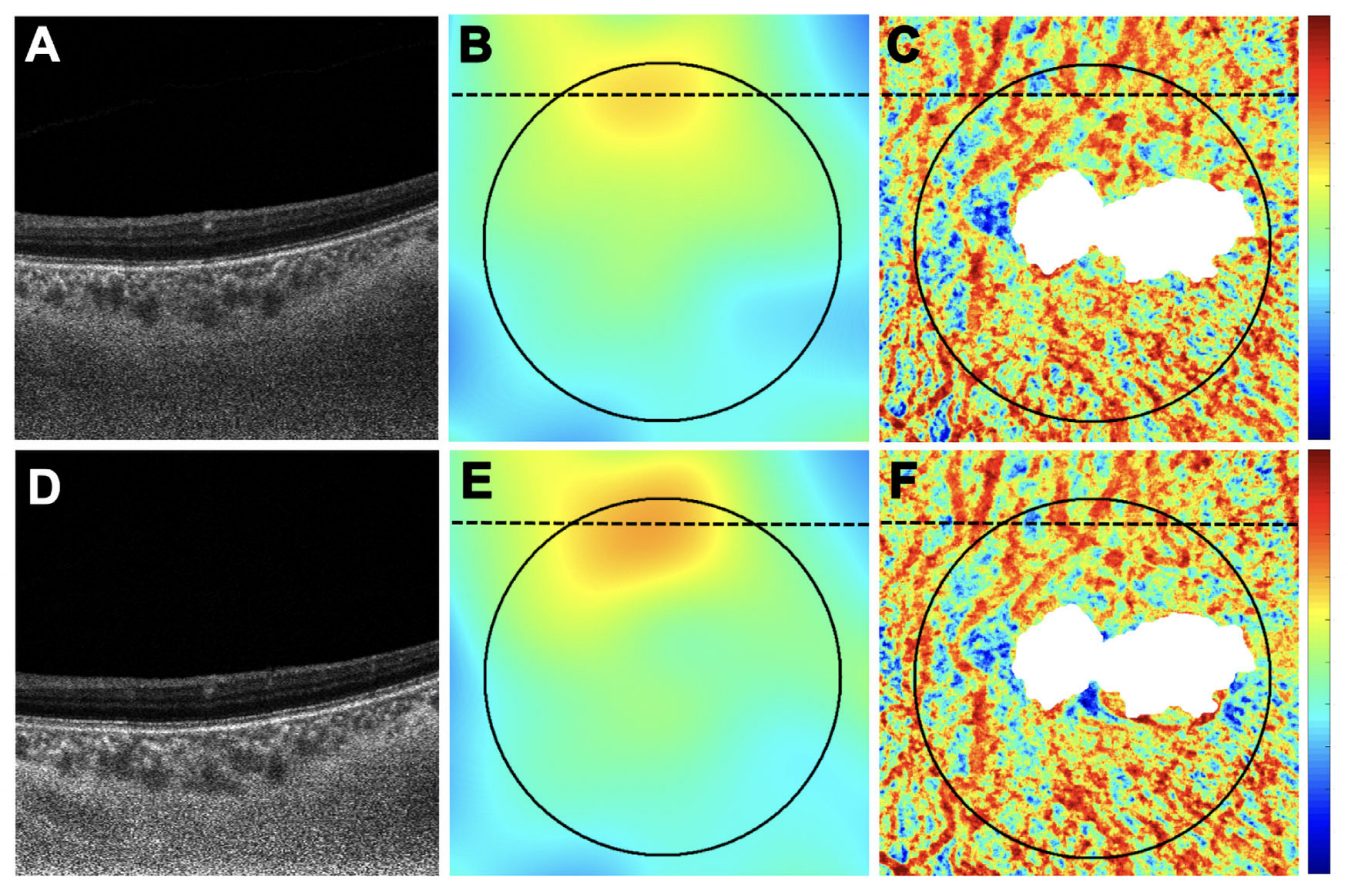
The same eye in Figure 7 with an increase in retinal PED volume and an increase in MCT measurement after anti-VEGF therapy. (**A**–**C**) Images are from the baseline visit before any treatment. (**D**–**F**) Images are 3 months after the baseline visit and after three monthly anti-VEGF injections. (**A**, **D**) B-scans after optical attenuation correction, corresponding to the *dotted lines* in **B**, **C**, **E**, and **F**. (**B**, **E**) MCT maps at baseline and after treatment. The 5-mm circle centered on the fovea is shown that was used to analyze the choroidal measurements from these scans. *Color bar:* 0 to 500 µm. MCT = 239.94 µm at baseline; MCT = 244.49 µm after treatment. (**C**, **F**) CVI maps at baseline and after treatment. *White mask* indicates the area that was excluded from the CVI measurement due to attenuation of the choroidal signal from the PED. Because the *white mask* accounts for 18% area of the 5-mm circle (>10%), this eye was excluded from CVI measurement. *Color bar:* 0 to 1.

There was no association between visual acuity and MCT measurements or between visual acuity and CVI measurements (all *P* ≥ 0.08). Among 33 PCV eyes with intraretinal fluid and/or subretinal fluid at baseline, 21 eyes achieved a dry macula after anti-VEGF therapy. However, there was no significant difference in MCT or CVI measurements between the eyes with a dry macula and those with fluid after treatment (all *P* ≥ 0.21) (Table 2). Four out of 34 PCV eyes (11.8%) had a type 2 MNV component. When comparing eyes with and without type 2 MNV, we found no significant difference in the measurements of baseline MCT, MCT after treatment, MCT change between visits, baseline CVI, or CVI change between visits (Table 3). However, after anti-VEGF injections, eyes with type 2 MNV had smaller CVI measurements compared with eyes without type 2 MNV (*P* = 0.022).

**Table 2.** MCT and CVI Measurements in Eyes With and Without Macular Fluid Before and After Treatment

	MCT (µm), Mean (SD)			CVI, Mean (SD)		
IRF/SRF After Treatment?	Before	After	Change	Before	After	Change
No (*n* = 21)^*^	221.3 (85.5)	207.0 (78.9)	14.3 (17.7)	0.599 (0.026)	0.601 (0.023)	0.003 (0.013)
Yes (*n* = 12)^*^	217.0 (67.9)	210.8 (74.0)	6.2 (17.7)	0.598 (0.021)	0.601 (0.021)	0.002 (0.011)
*P* ^†^	0.88	0.89	0.21	0.97	0.96	0.98

* Two-sample *t*-test.

† For the CVI, *n* = 19 for no and *n* = 10 for yes.

**Table 3.** MCT and CVI Measurements in Eyes With and Without Type 2 MNV Before and After Treatment

	MCT (µm), Mean (SD)			CVI, Mean (SD)		
Type 2 MNV Component?	Before	After	Change	Before	After	Change
No (*n* = 30)	227.4 (83.4)	216.5 (79.4)	10.9 (17.5)	0.602 (0.024)	0.606 (0.023)	0.004 (0.014)
Yes (*n* = 4)	190.0 (27.6)	168.4 (15.4)	21.6 (22.8)	0.581 (0.003)	0.581 (0.012)	0.000 (0.011)
*P* ^*^	0.45	0.20	0.34	0.094	0.022	0.66

* Mann–Whitney test.

## Discussion

In this study, we found that both MCT and PED volume measurements decreased in eyes with PCV after anti-VEGF therapy. The decrease in PED volume measurements was correlated with the decrease in MCT measurements and the increase in CVI measurements. To the best of our knowledge, our study is the first to measure the MCT and CVI in eyes with PCV in a 6 × 6-mm volumetric scan and correlate them with the change in PED volume. Moreover, we were cautious when assessing the MCT and CVI measurements due to the choroidal signal attenuation that occurred under PEDs, so we excluded regions where the BM to choroidal/scleral interface and choroidal vessels could not be clearly detected due to signal blockage resulting from the overlying large PEDs, which has been a challenge when performing choroidal analyses in eyes with PCV. Moreover, this limitation has been neglected in other studies.

**Figure 9.**
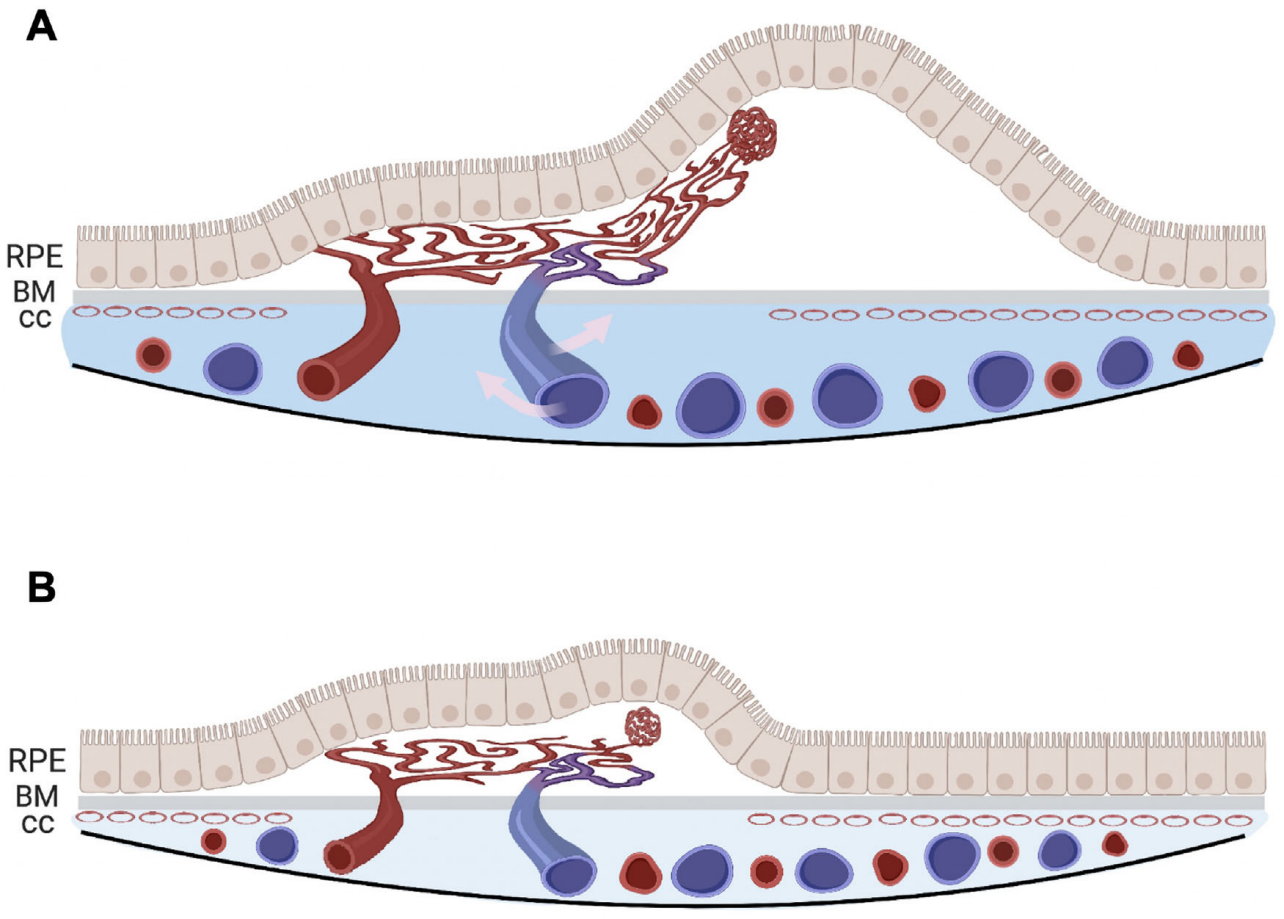
A schematic showing the change in the choroid of an eye with PCV and its association with the decrease in retinal PED volume after anti-VEGF therapy. (**A**) At baseline, the PCV lesion serves as a high-volume arteriovenous shunt between the choroidal arterial and venous circulations. The higher hydrostatic pressure in the venous system from this shunt results in transudation of fluid, as well as the fibrin and blood plasma, causing an increase in the volume of the choroidal stroma. The PED results from the VEGF-mediated exudation, as well as the transudation of fluid under the RPE from this arterialized high-flow lesion. (**B**) After anti-VEGF therapy, the VEGF-mediated exudation and blood flow through the vascular shunt are reduced. Because the choroidal venous volume mainly accounts for the choroidal thickness, the choroidal thickness decreases when the venous volume decreases after treatment. In addition, the CVI increases after treatment because the excess stromal transudation is resorbed. Compared with the decrease in the choroidal vascular volume, the choroidal stromal volume decreases to a greater extent, which results in an increase in the ratio of the choroidal vascular volume to the total choroidal volume (CVI). Finally, less exudation from the neovascular lesion and less transudation in the choroid after treatment will contribute to less fluid and lower pressure under the RPE, resulting in the decrease in PED volume. CC, choriocapillaris.

Several studies have used a single-raster B-scan and concluded that the subfoveal choroidal thickness decreased in eyes with PCV after anti-VEGF treatment.^23^^,^^24^ Previous explanations for this phenomenon have suggested that this change was due to anti-VEGF therapy with the assumption that the drugs were acting directly on the choroidal vasculature. Other factors that may affect the change in MCT include diurnal variation in blood pressure and certain systematic medicine.^25^^,^^26^ Here, we provide an alternative explanation for why the choroidal thickness changes in PCV after treatment (Fig. 9). Because it is known that the choriocapillaris is a dense, terminal capillary bed for the entire choroidal circulation, we propose that, as PCV progresses, extensive choriocapillaris impairment and loss occur, as shown in specimens from surgical excision of PCV lesions.^27^ The PCV lesion then serves as an alternative shunt between choroidal arterial and venous circulation, also known as an arteriovenous shunt. The situation may be similar for other type 1 and type 2 neovascular lesions arising from the choroid, but PCV may be the neovascular lesion arising from the choroidal circulation with the lowest resistance and the highest flow. However, when compared with the normal choriocapillaris vascular bed, the PCV lesion can be considered a lower resistance shunt with a higher blood volume, and this shunt would direct more blood into the choroidal venous circulation, resulting in dilated choroidal veins. Because the choroidal thickness is mostly determined by the choroidal vascular volume,^22^ this would result in an increased choroidal thickness. In addition, the higher intraluminal hydrostatic pressure within the choroidal venules could result in increased transudation into the choroid. After anti-VEGF therapy, the VEGF-mediated exudation from the neovascular lesion is reduced and blood flow through the vascular shunt is reduced. Because the choroidal venous volume mainly accounts for the choroidal thickness, the choroidal thickness could decrease when the venous volume decreases after treatment, and the choroidal thickness might decrease even more with a reduction in abnormal transudation of fluid into the choroidal stroma and a resorption of excess stromal fluid. Thus, the MCT would decrease after anti-VEGF therapy, whereas the CVI, which is usually constant across age and macular region,^22^ might change depending on the relative changes in the choroidal vascular volume and choroidal stromal volume.

As for the CVI in PCV eyes, Lee et al.^28^ found that PCV eyes had lower CVIs compared with normal controls at baseline. This would suggest that the choroidal vascular volume is lower compared with normal eyes or the choroidal stromal volume is increased. In nine fellow eyes with newly developed PCV, a decrease in CVI was observed when compared with CVI at baseline. The authors proposed that the reduction in choriocapillaris density and choroidal vascular diameter could possibly explain low CVI, which appears to be contradicted by the fact that the dilated choroidal vessels can be observed on both ICGA and OCT. A more likely explanation is that the increased choroidal hyperpermeability seen in eyes with PCV would increase the choroidal stroma volume due to transudation of fluid from the increased choroidal hyperpermeability.^29^ In PCV eyes, this choroidal hyperpermeability seen on ICGA is believed to result from an increase in fluid and lipoprotein extravasation from the choriocapillaris and larger choroidal vessels into the surrounding choroidal stroma.^30^ This angiographic finding is consistent with the histopathologic observation of massive exudation of fibrin and blood from dilated hyalinized choroidal vessels in PCV specimens.^27^ In Figure 9, we combined our results with previously published ICGA results to speculate that, at baseline, the PCV lesion serves as a choroidal arteriovenous shunt, resulting in higher hydrostatic pressure within the venous system that leads to transudation of fluid which causes an increase in the volume of the choroidal stroma. The CVI increase after treatment can be explained by the resorption of the excess stromal transudation. To explain an increased CVI, the decrease in the choroidal stromal volume would have to be relatively greater than the decrease in choroidal vascular volume, resulting in an increase in the ratio of the choroidal vascular volume to the total choroidal volume (CVI). Of note, a previously published study following the recurrence of PCV in eyes after anti-VEGF treatment showed that an increase in choroidal vessel diameter was found to precede the change in central choroidal thickness,^31^ which would be consistent with our model of the PCV lesion serving as an arteriovenous shunt with increasing blood flow through the neovascular lesion prior to the onset of recurrent exudation, and this enlargement of the choroidal vessels would contribute to the increase in choroidal thickness in PCV. These observations suggest that excess transudation of fluid into the choroidal stroma in eyes with PCV deserves greater attention, and it can be followed using the CVI measurements obtained from SS-OCT imaging without the need to demonstrate choroidal hyperpermeability using ICGA.

In our study, although there was no significant difference in MCT or CVI measurements between the eyes with dry maculae and those with fluid after treatment, we did find that a decrease in PED volume was associated with a decrease in MCT and an increase in CVI measurements. In a PCV study using pro re nata anti-VEGF therapy, Chan et al.^32^ concluded that increases in the PED volume at one visit, despite dry macula, are associated with retreatment at the next visit. The predictive value of PED volume assessment was also observed in eyes with non-PCV MNV.^33^^,^^34^ These observations emphasize the predictive importance of PED volume measurements in the management of PCV and non-PCV choroidal neovascular lesions. Here, we provide a mechanism for the development of PED and its resolution. As shown in Figure 9, PED results from the increased hydrostatic pressure beneath the RPE due to the VEGF-mediated exudation from the neovascular lesion and from the increased transudation of fluid into the choroidal stroma. After treatment, a decrease in exudation from the neovascular lesion and a decrease in transudation into the choroidal stroma would lower the hydrostatic pressure under the RPE, resulting in a decrease of PED volume. The opposite would occur when the effect of anti-VEGF therapy wanes on the neovascular lesion; increased flow occurs through the arteriovenous shunt, and the PED enlarges.

Our study is limited by the small sample size, the variable follow-up intervals, and different types of anti-VEGF medicine used. However, we are encouraged by our results, and, given the statistical significance of our findings, we anticipate that a prospective study with well-defined follow-up intervals and standardized treatments would confirm and strengthen our results. Also, 12 × 12-mm scan patterns were not routinely used at both institutions during the time these patients were imaged. To include as many patients as possible and maintain consistency, we included only the PCV eyes imaged with 6 × 6-mm scans and analyzed only the 6 × 6-mm images for PED volume and choroidal measurements. It remains to be determined whether the choroidal changes extend beyond the 6 × 6-mm scan area and whether these changes are predictive of long-term visual function, recurrence of exudation, PED volumes, and future treatments. Additional studies with longer follow-up will help answer these questions. Of note, variability in the lateral scale of the images, particularly in the absence of axial length corrections, can introduce errors in the exact PED and choroidal measurements.^35^ However, this limitation should not significantly affect the conclusions of our study, as we compared the longitudinal changes before and after treatment in the same eye. In addition, signal attenuation due to the PEDs prevented us from quantifying the choroidal vessels under the PEDs in certain cases. It is possible that the CVI of the choroid underlying a PED differs from that elsewhere in the choroid within the 5-mm circular region investigated, but this type of localized variability within a 5-mm circular region seems unlikely due to the interconnectivity of the choroidal vasculature. This relationship between the changes in the choroid and the PED volumes should be explored in the future by following these eyes as the PED volumes increase and decrease. Finally, there may be a correlation of change in SRF/IRF volume with the change in choroidal measurements, which should be explored with reliable algorithms to quantitate SRF/IRF volume, especially when type 2 MNV presents.

In conclusion, the MCT and PED volume measurements in treatment-naïve PCV eyes decreased after anti-VEGF therapy. The decrease in PED volume was correlated with the decrease in MCT measurements and the increase in CVI measurements. We propose that, at baseline, the PCV lesion serves as a high-volume arteriovenous shunt between the choroidal arterial and venous circulations, causing an increase in choroidal stromal transudation and a dilation of choroidal venous circulation. After treatment, the blood flow through the vascular shunt is reduced, the excess stromal transudation is resorbed, and the exudation from the neovascular lesion is reduced, as well, resulting in a thinning of the choroid, an increase in the CVI, and resolution of the PED. By using SS-OCT to image the choroid in eyes with PCV, we acquired depth-resolved measurements that complement the observations from ICGA and provide a basis for understanding the hemodynamic changes that occur in PCV.
